# Assessment at UK medical schools varies substantially in volume, type and intensity and correlates with postgraduate attainment

**DOI:** 10.1186/s12909-015-0428-9

**Published:** 2015-09-11

**Authors:** Oliver Patrick Devine, Andrew Christopher Harborne, I. C. McManus

**Affiliations:** 1 Division of Infection & Immunity, University College London, London, WC1E 6BT UK; 2 School of Medicine, David Weatherall Building, Keele University, Stoke-on-Trent, Staffordshire, ST5 5BG UK; 3 UCL Medical School, University College London, London, WC1E 6BT UK

## Abstract

**Background:**

In the United Kingdom (UK), medical schools are free to develop local systems and policies that govern student assessment and progression. Successful completion of an undergraduate medical degree results in the automatic award of a provisional licence to practice medicine by the General Medical Council (GMC). Such a licensing process relies heavily on the assumption that individual schools develop similarly rigorous assessment policies. Little work has evaluated variability of undergraduate medical assessment between medical schools. That absence is important in the light of the GMC’s recent announcement of the introduction of the UKMLA (UK Medical Licensing Assessment) for all doctors who wish to practise in the UK. The present study aimed to quantify and compare the volume, type and intensity of summative assessment across medicine (A100) courses in the United Kingdom, and to assess whether intensity of assessment correlates with the postgraduate attainment of doctors from these schools.

**Methods:**

Locally knowledgeable students in each school were approached to take part in guided-questionnaire interviews via telephone or Skype^TM^. Their understanding of assessment at their medical school was probed, and later validated with the assessment department of the respective medical school. We gathered data for 25 of 27 A100 programmes in the UK and compared volume, type and intensity of assessment between schools. We then correlated these data with the mean first-attempt score of graduates sitting MRCGP and MRCP(UK), as well as with UKFPO selection measures.

**Results:**

The median written assessment volume across all schools was 2000 min (mean = 2027, SD = 586, LQ = 1500, UQ = 2500, range = 1000–3200) and 1400 marks (mean = 1555, SD = 463, LQ = 1200, UQ = 1800, range = 1100–2800). The median practical assessment volume was 400 min (mean = 472, SD = 207, LQ = 400, UQ = 600, range = 200–1000). The median intensity (minutes per mark ratio) of summative written assessment was 1.24 min per mark (mean = 1.28, SD = 0.30, LQ = 1.11, UQ = 1.37, range = 0.85–2.08). An exploratory analysis suggested a significant correlation of total assessment time with mean first-attempt score on both the knowledge and the clinical assessments of MRCGP and of MRCP(UK).

**Conclusions:**

There are substantial differences in the volume, format and intensity of undergraduate assessment between UK medical schools. These findings suggest a potential for differences in the reliability of detecting poorly performing students, or differences in identifying and stratifying academically equivalent students for ranking in the Foundation Programme Application System (FPAS). Furthermore, these differences appear to directly correlate with performance in postgraduate examinations. Taken together, our findings highlight highly variable local assessment procedures that warrant further investigation to establish their potential impact on students.

**Electronic supplementary material:**

The online version of this article (doi:10.1186/s12909-015-0428-9) contains supplementary material, which is available to authorized users.

## Background

Over the past decade or more there has been a growing public scrutiny of standards within the medical profession, primarily at postgraduate level but more recently at undergraduate level. While local assessment of medical students by individual schools has traditionally offered a valuable tool for ensuring that the expected standards are being met prior to employment within the NHS, the UK lacks a truly standardised system capable of comparing the performance of students between schools. The General Medical Council (GMC) has recently announced that it wishes to work towards a national system of assessment, the UK Medical Licensing Assessment (UKMLA) for all doctors, including UK graduates, who wish to practise medicine in the UK, although it is unlikely to be introduced for UK graduates before 2021 at the earliest [1]. At present, standards at individual schools are assured by the Quality Assurance Agency’s (QAA) external examiner system and GMC Quality Assurance of Basic Medical Education (QABME) inspections. Successful completion of an undergraduate medical degree, therefore, automatically results in a provisional license to practice medicine being granted by the GMC [2]. This process of licensing relies heavily on the assumption that individual schools are both sufficiently and similarly rigorous in assessing the standard of their students.

Although the QABME and external examiners system scrutinises undergraduate assessment on a regular basis, their reports and recommendations are qualitative in nature, and do not quantitatively compare assessment policies between medical schools [3]. Efforts have been made to rectify this. The Medical Schools Council Assessment Alliance (MSC-AA) was setup as a collaborative effort to incorporate a shared bank of questions as a proportion of the local final year examinations at each medical school. In principle such a question bank enables quantitative comparison of student performance between schools, however the MSC-AA is yet to report on any such comparison [4] and there are potentially many practical and theoretical problems in achieving that aim.

After graduation, newly qualified UK doctors work for 2 years in approved Foundation Posts (F1 and F2) overseen by Foundation Schools, where they rotate around a number of different specialties, including General Practice in some cases. Recent reforms to the Foundation Programme Application System (FPAS) have sought to make the Educational Performance Measure (EPM) more fine-grained, using a decile-based rank rather than a quartile-based rank of student performance. Despite these changes, the EPM remains a norm-referenced system which of necessity is insensitive to variation in entry qualifications between medical schools or between cohorts within medical schools. The revised EPM with deciles puts increased emphasis on student performance during medical school in the context of a system where the medical schools internally assess and rank students based on local policies. A concern is that two equally able graduates may score differently on their EPM simply because of variability in local assessment procedures and policies. Overseas, countries like the United States and Canada overcome concerns of local variation in assessment policies through systems of national multistep licensing examinations that must be passed before medical graduates are able to work independently as doctors [5, 6]. These systems also enable fair ranking of students for highly competitive postgraduate training posts.

In 2005 the GMC undertook a formal consultation on the matter of introducing a national exit examination. This lead to the publication of the Strategic Proposals for Assessment [7]. In that publication, the GMC called for improvements to the external examiner system and a review of the QABME process and indicated that they would “look into the policy implications of shared questions or a national examination” [7]. Recently, Health Education England’s medical director, Professor Wendy Reid, published a report proposing “full GMC registration should be brought forward to the point of graduation” with the possibility of “a national examination (taken by all applicants – i.e. from UK, European Economic Area (EEA) and overseas medical schools). This would allow applicants to be ranked for the purpose of allocation to the number of Foundation places required” [8]. In September 2014, the GMC publicly announced its aspiration to develop a single national licensing exam for all UK doctors, although the legal basis of the GMC imposing such an examination on EEA doctors has been questioned and the precise nature of the licensing examination remains poorly defined [9]. As mentioned above, the development of UKMLA was announced in 2015.

Whilst debates have taken place nationally, student opinion on a national licensing examination remains mixed, with the BMA Medical Students Committee (BMA-MSC) having been publicly opposed [10]. Despite this, a national survey of final year medical students suggests that students would be in favour of such a system [11]. Student stakeholders argue that local examinations preserve diversity in medical education with translational benefits to the NHS workforce. They also suggest that a national system of assessment risks undergraduate programmes ‘teaching for the exam’ with students adopting increasingly competitive attitudes towards their peers [12]. These remarks echo the sentiments of the GMC who have historically argued that a national exit examination cannot adequately substitute multiple years of cumulative assessment conducted by individual schools [7].

Whilst a system of national assessment might provide a more defensible way to rank students for Foundation Programme jobs, it also has the opportunity to clearly define a level of expected undergraduate medical knowledge. This would be especially true of a multistep system such as the USMLE which interrogates basic science and clinical knowledge across four independent examinations. A frequently cited axiom in medical education is that ‘assessment drives learning’, and there are empirical studies to that effect [13–15]. It would, therefore, be important in designing such a system to understand whether more learning takes place at medical schools prescribing greater amounts of assessment, and if that in turn results in the acquisition of more ‘medical capital’ [16] that is later advantageous to candidates sitting postgraduate medical examinations. By such logic, standardising the amount of assessment could raise the standard of postgraduate knowledge, as measured by improved performance on postgraduate assessments. This could be an achievable goal of a national licensing system if it imposed a sufficient volume of assessment.

In the context of ongoing debates regarding a national examination system and its nature, it is important to examine the extent of diversity that currently exists in undergraduate medical school assessment in order to evaluate the fundamental need for standardisation. Anecdotal evidence and qualitative studies have found differences in finals examinations between schools [3] and differences in the passing scores set for the same objective examinations at different schools [17]. A number of studies have also found that performance in postgraduate examination depends on the medical school from which a doctor graduates [18]. To provide an evidence base for discussions on this topic, we sought to quantify and compare the variability of summative assessment volume and intensity across each undergraduate medical course in the UK. We also carried out an exploratory study correlating undergraduate assessment volume with postgraduate examination performance.

## Methods

We approached data collection concerning assessment in undergraduate medical schools in an incremental way. First, student representatives from all UK medical schools were invited to take part in individual telephone/Skype™ interviews to gain contextual insight into the assessment process at each medical school. Given the variety and complexity of undergraduate medical assessment, the interview consisted of a guided-questionnaire that allowed us to ask detailed questions about assessment in the most recent academic year, whilst facilitating real-time clarification. Interviews were conducted by two researchers (ACH + OPD), one of whom worked through the questionnaire with the student (ACH), whilst the other recorded the responses onto a data sheet (OPD). Quantitative data were then validated through direct communication with the respective medical schools.

Our data collection identified key information about the school in question (course length, compulsory intercalation status) as well as year-by-year quantitative data regarding written and practical assessments (e.g. OSCEs). UCAS (Universities and Colleges Admission Service) uses codes of A100, A101, A102 and A104 for various types of medical course. We examined the conventional 5- or 6-year undergraduate medical courses (A100) across all UK universities, excluding graduate-entry 4-year (A101), 6-year widening access (A102) and 6-year science foundation year (A104) courses. We aimed to quantify summative, timed assessments across all “A100” courses in the UK. Key outcomes focused on comparative indices of total written assessment volume (in terms of minutes and raw marks), total volume of practical assessment (in terms of minutes of assessment) and intensity of assessment (calculated as minutes per raw mark). A ‘raw mark’ is defined in this study as the smallest unadjusted indivisible unit of award that a student may achieve during a written assessment – examples include an individual Single Best Answer (SBA)/Multiple Choice Question (MCQ), an individual component of an Extended Matching Question (EMQ), or an individual mark point as part of a free-text answer (e.g. short answer question (SAQ) or essay question).

The Medical Schools Council describes 33 medical schools providing undergraduate training [19]. Three of these are relatively new schools (Durham, Lancaster and Swansea), one (St Andrews) only teaches pre-clinical medicine, and two (Exeter and Plymouth) have only recently been formed by the splitting of Peninsula Medical School. We therefore contacted the remaining 27 medical schools that are offering A100 programmes and have been running for at least 5/6 years. Fifty-six percent of student representatives (15/27) were able to provide some information regarding assessment at their respective schools. No student representatives were able to provide us with sufficient information about their assessment system for our analysis, but three were able to do so after referring to course documentation. After direct communication with medical school assessment teams, ninety-three percent (25/27) of A100 programmes provided us with data that were sufficient for analysis. Norwich Medical School was able to provide some data but these were insufficient for our analysis deadline. The University of Leicester did not respond to participation requests. Five schools had complete information on minutes of assessment but not marks, for these schools, only minutes were included in our analysis. In a few cases when an item of assessment could not be validated or was marked according to an arbitrary marking scheme, we applied a school’s standard marks per minute ratio to determine a suitable raw mark based on a validated length of time for the assessment item in question. Data pertaining to the USMLE were obtained from the website of the examinations [20].

For convenience we will refer to years 1 and 2 of courses as ‘preclinical’, and will refer to final 3 years of a course as ‘clinical’. In doing so we recognize that some schools run fully integrated courses, whereas others still have a very traditional approach emphasizing basic medical sciences in the early years. We also recognize that some schools have a compulsory intercalated or integrated studies year (e.g. Oxford, Cambridge, Imperial, UCL, and Nottingham), and we have not considered assessment in those or any other intercalated years. A minority of schools used some sequential testing methods (i.e. all students would sit Part A of an examination, and only those with low marks would sit a Part B examination to assess whether they passed or failed) we included data solely for the examination sat by *all* students at the institution (e.g. Part A) as that gives a fair indication of the assessment load of a typical student.

### Postgraduate attainment

On an exploratory basis we related assessment volume at medical school to postgraduate performance at MRCP(UK) and MRCGP. MRCP(UK) is an ‘entry examination’ for doctors wanting to train as physicians, typically taken within 2 to 3 years of qualification, and consists of three parts, Part 1 and Part 2 which are MCQ-based knowledge tests, and PACES which is an OSCE-style clinical assessment of physical examination (in real patients) and communication skills (in simulated patients) [21–23]. MRCGP is an exit examination taken towards the end of postgraduate training in General Practice, typically 4 to 5 years after qualification. MRCGP AKT (Applied Knowledge Test) is an MCQ-based knowledge assessment and MRCGP CSA (Clinical Skills Assessment) is an OSCE-style simulated surgery concentrating mainly on communication skills [24]. Candidates who have taken both MRCP(UK) and MRCGP attain similarly in knowledge and skills domains [25]. Published data were available for the mean mark attained by graduates of UK medical schools at the MRCGP AKT (knowledge) and CSA (clinical) assessments [26] from 2008 to 2013, and the MRCP(UK) Part 1 and Part 2 (knowledge) and PACES (clinical) assessments from 2002 to 2013 [26]. For all assessments, marks were considered only at first attempts, as is conventional. Data for MRCGP were available separately for the London medical schools, but for MRCP(UK) were only available for all London schools combined. Data were only analysed for established medical schools and not the more recently established schools, making samples much larger and mean scores more stable. Results of postgraduate assessments are expressed as percentage marks from the pass mark (which varied from diet to diet), and then converted to percentile ranks for averaging, as is conventional in studies of postgraduate education [25, 27, 28].

### Entry qualifications

Entry qualifications differ between medical schools, and it is probable that they correlate with postgraduate qualifications. They may also relate to assessment volume. Mean tariff scores calculated by UCAS are available for UK medical schools at www.thecompleteuniversityguide.co.uk/league-tables/rankings?s=Medicine (‘Entry standards’), along with summaries of data on Student Satisfaction and Research Assessment. Data are described as ‘2015’ (i.e. the most recent data available for those applying for entry in October 2015) but in fact are based on Higher Education Statistics Agency (HESA) data for 2012–13. Measures of student satisfaction (based on final year students in the National Student Survey (NSS) for 2013) and research intensity (based on the 2008 Research Assessment Exercise (RAE) are also taken from the same source).

### UKFPO results

The UK Foundation Programme Office (UKFPO) publishes results for its Situational Judgment Test (SJT) and its Educational Progress Measure (EPM) (http://www.foundationprogramme.nhs.uk/download.asp?file=Stats_and_facts_FP2014_interim_report_4_April_2014_FINAL.pdf), as average values for each medical school, and we have included those results for 2014 in the present analyses.

### Statistical analyses

After validation, data were entered and stored in an Excel spreadsheet. Data were analysed using GraphPad Prism 6.0, and were also imported into IBM SPSS 22.0. Analysis of scattergrams (see below) suggested that there might be occasional outliers in the data, and therefore inferential statistics used Spearman correlations (*r*_*S*_), which are non-parametric, with partial correlations calculated in the conventional way but using *r*_*S*_ rather than Pearson correlations.

### Ethical approval

The nature of the study was presented at several stages to the UCL Research Ethics Committee and it was agreed that the study conducted was exempt from the requirement to obtain ethical approval (http://ethics.grad.ucl.ac.uk/exemptions.php).

## Results

### Volume of summative, timed assessment varies substantially between schools

**Fig. 1:**
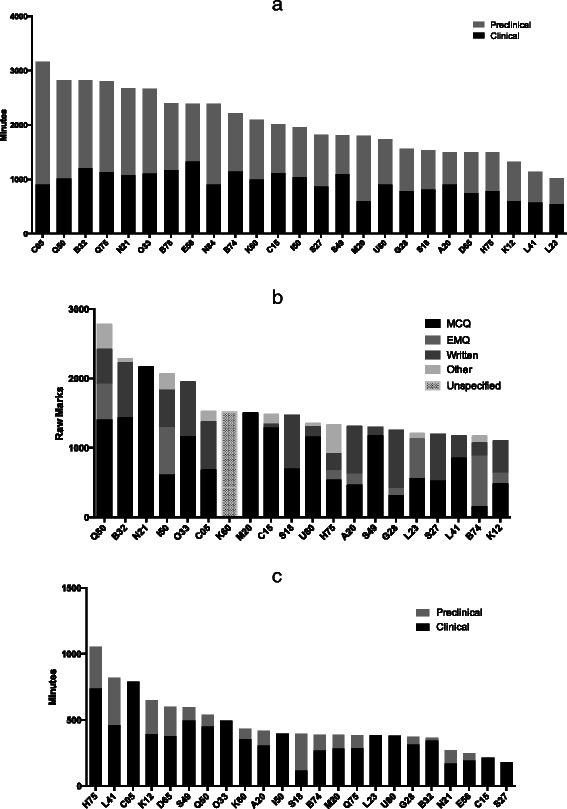
Total volume of summative, timed assessment experienced by students during a UK medical degree programme. Measured in total minutes (**a**), raw marks (**b**) and total minutes of practical examination (**c**). Labels represent UCAS institution codes for individual medical schools

We define assessment volume in terms of total minutes of assessment and total number of raw marks across the entirety of a medical degree. The median volume of written assessment across an entire medical course was 1900 min (Fig. 1a; mean = 2000, SD = 600, LQ = 1500, UQ = 2400, range = 1000–3200) and 1500 marks (Fig. 1b; mean = 1600, SD = 500, LQ = 1200, UQ = 1600, range = 1100–2800). Notably, there is greater variation in the volume (in minutes) of written assessment in preclinical years (SD = 500) as compared to clinical years (SD = 300). Volume (in minutes) of practical assessment (e.g. OSCEs) also varies substantially between schools (Fig. 1c; median = 400, mean = 500, SD = 200, LQ = 400, UQ = 600, range = 200–1100).

**Fig. 2:**
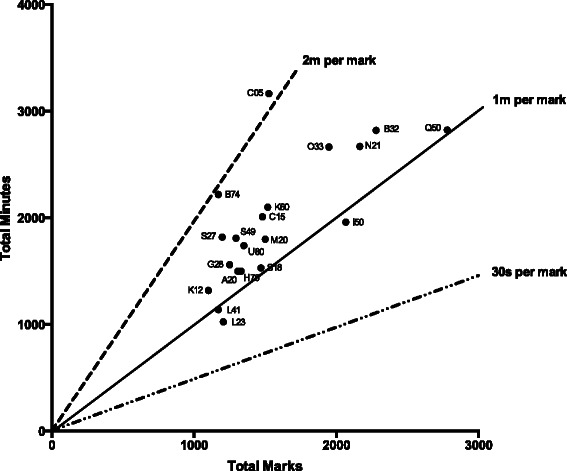
Scattergram demonstrating the relationship between total raw marks and total minutes of summative, timed, written assessment over the length of the entire medical degree. The three diagonal lines show 2 min per mark, 1 min per mark and 30 s per mark. Labels represent UCAS institution codes for individual medical schools

As expected, there is a positive correlation between total raw marks and total minutes of assessment over the length of an undergraduate medical course (*r*_*S*_ =0.75, *n* = 17, *p* = .0005; Fig. 2). Since total assessment time is the more objective, more easily quantified measure, we use it in the correlation analyses below. However correlations with total raw marks are also reported in Additional file 1.

The composition of assessment formats also varies substantially between schools (Fig. 1b), with schools such as St George’s (University of London) relying almost entirely on MCQ/SBA. The University of Glasgow, on the other hand, seldom uses the MCQ/SBA format, despite those schools having similar numbers of raw marks available (Table 1).

**Table 1:** Volume of written and practical assessment at UK medical schools

					Summative, Timed, Written Assessment							Practical Assessment		
					*Minutes*			*Marks*			*Intensity*	*Minutes*		
	School	UCAS Code	Course Length	Compulsory Intercalation	Pre-clinical	Clinical	Total	Pre-clinical	Clinical	Total	min/ mark	Pre-clinical	Clinical	Total
1	Brighton & Sussex Medical School	B74	5	N	1100	1100	2200	400	800	1200	1.9	100	300	400
2	Cardiff University	C15	5	N	900	1100	2000	700	800	1500	1.36	0	200	200
3	Hull and York Medical School	H75	5	N	700	800	1500	800	500	1300	1.13	300	700	1000
4	Imperial College London	I50	6	Y	900	1000	1900	1100	1000	2100	0.95	0	400	400
5	Keele University	K12	5	N	700	600	1300	600	500	1100	1.20	300	400	700
6	King’s College London	K60	5	N	1100	1000	2100	900	600	1500	1.38	100	400	500
7	Newcastle University	N21	5	N	1600	1100	2700	1300	800	2100	1.23	100	200	300
8	Queen Mary, University of London	Q50	5	N	1800	1000	2800	1900	900	2800	1.02	100	500	600
9	Queen’s University Belfast	Q75	5	N	1700	1100	2800	?	?	?	?	100	300	400
10	St George’s, University of London	S49	5	N	700	1100	1800	500	800	1300	1.40	100	500	600
11	The University of Edinburgh	E56	5	N	1100	1300	2400	?	?	?	?	100	200	300
12	The University of Sheffield	S18	5	N	700	800	1500	700	800	1500	1.04	300	100	400
13	University College London	U80	6	Y	800	900	1700	600	800	1400	1.29	0	400	400
14	University of Aberdeen	A20	5	N	600	900	1500	500	800	1300	1.15	100	300	400
15	University of Birmingham	B32	5	N	1600	1200	2800	1500	800	2300	1.24	0	300	300
16	University of Bristol	B78	5	N	1200	1200	2400	?	?	?	?	?	?	?
17	University of Cambridge	C05	6	Y	2300	900	3200	900	600	1500	2.08	0	800	800
18	University of Dundee	D65	5	N	800	700	1500	?	?	?	?	200	400	600
19	University of Glasgow	G28	5	N	800	800	1600	500	700	1200	1.25	100	300	400
20	University of Leeds	L23	5	N	500	500	1000	600	600	1200	0.85	0	400	400
21	University of Liverpool	L41	5	N	600	600	1200	600	600	1200	0.97	400	500	900
22	University of Manchester	M20	5	N	1200	600	1800	1000	500	1500	1.20	100	300	400
23	University of Nottingham	N84	5	Y	1500	900	2400	?	?	?	?	?	?	?
24	University of Oxford	O33	6	Y	1600	1100	2700	900	1100	2000	1.37	0	500	500
25	University of Southampton	S27	5	Y	1000	900	1900	600	600	1200	1.52	0	200	200

Data are divided into preclinical (years 1 and 2) and clinical (years 3, 4 and 5). Minutes and marks are rounded to the nearest hundred

^*?*^ *- Indicates data that schools were unwilling or unable to validate or provide*

### Assessment intensity varies substantially between medical schools

In addition to comparing the volume of assessment, we were also interested in the time–pressure or ‘intensity’ of assessment. Figure 2 demonstrates the correlation between total marks and total minutes of summative, timed, written assessment prescribed by the schools included in our analysis. The median intensity of assessment in our analysis was 1.24 min per mark (mean = 1.28, SD = 0.30, LQ = 1.11, UQ = 1.37, range = 0.85–2.08). The majority of schools prescribe between 1 and 2 min per mark, although notable exceptions do exist.

### Assessment volume correlates with postgraduate performance

Outcome measures were available for five postgraduate examinations, the AKT and CSA assessments of MRCGP, and Part 1, Part 2 and PACES assessments of MRCP(UK). Correlations of the five assessments with each other, and with other variables in the study are shown in detail in Additional files 1 and 2. The mean *r*_*S*_ between the five postgraduate assessments was .826 (median = .816, n correlations = 10, range = .645–.973), suggesting that they are all measuring a similar construct (and that is supported by other analyses at the level of the individual which shows high correlations between MRCP(UK) and MRCGP marks [25]). For simplicity we therefore converted all postgraduate marks to percentile ranks and calculated the mean rank across all of the five assessments. We refer to this measure as mean postgraduate attainment.

**Fig. 3:**
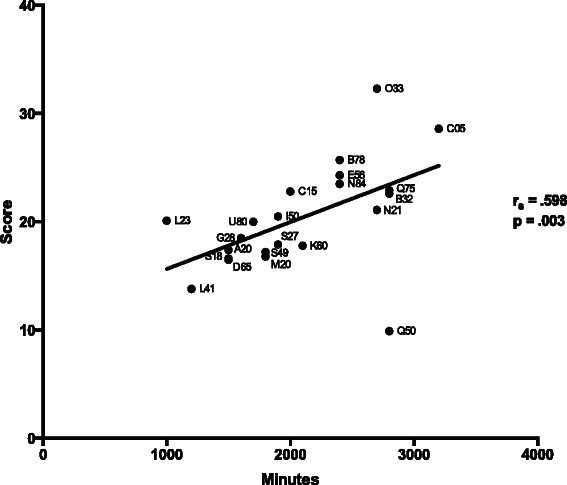
Scattergram demonstrating the correlations between minutes of written assessment at a particular school and mean first-attempt scores for graduates sitting the MRCGP AKT examinations. Labels represent UCAS institution codes for individual medical schools

Overall there was a significant correlation between total minutes of assessment time and mean postgraduate attainment (*r*_*S*_ = .515, *p* = .014, *n* = 22). An example scattergram is shown in Fig. 3 for the relationship between MRCGP AKT and minutes of assessment time (*r*_*S*_ = .598, *n* = 22, *p* = .003) (Fig. 3). Queen Mary (University of London) appeared to be an outlier, and was responsible for our decision to use Spearman correlations for the analyses. Removing Queen Mary (University of London) gave a higher overall correlation of total minutes of assessment time and mean postgraduate attainment (*r*_*S*_ = .701, *p* = .0004, *n* = 21). However, there was no theoretical reason for removing this medical school from the analyses, and therefore we decided to include it and use non-parametric statistics for all analyses.

A notable feature of Fig. 3 is that Oxford and Cambridge have higher postgraduate attainment and relatively more assessment than other medical schools. Oxford and Cambridge also have higher entry qualifications than other medical schools, and it is possible that that is responsible for the relationship shown in Fig. 3. Removing Oxford and Cambridge as well as Queen Mary (University of London) left the correlation as significant (*r*_*S*_ = .633, *n* = 19, *p* = .004), although including Queen Mary (University of London) meant that the correlation did not quite achieve significance (*r*_*S*_ = .417, *n* = 20, *p* = .067).

**Fig. 4:**
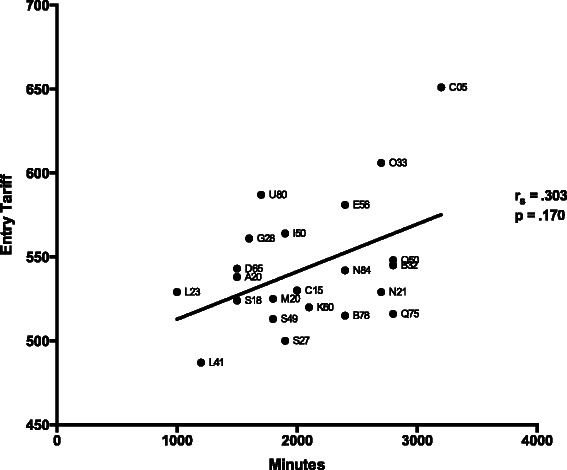
Scattergram of minutes of written assessment in relation to entry qualification (mean UCAS tariff points). Labels represent UCAS institution codes for individual medical schools

The proper way to assess the effect of entry qualifications is to assess the partial correlation of mean postgraduate attainment with minutes of assessment, after taking entry qualifications into account. The following correlations were carried out using all medical schools, including Oxford, Cambridge and Queen Mary (University of London). Simple correlations showed, unsurprisingly, that schools with higher entry qualifications had higher postgraduate attainment (*r*_*S*_ = .447, *n* = 22, *p* = .037). However the simple correlation of minutes of assessment time with entry qualifications, albeit positive, was not significant (*r*_*S*_ = .303, *n* = 22, *p* = .170); see Fig. 4. The partial correlation of mean postgraduate attainment with minutes of assessment time, after taking entry qualifications into account remained significant (partial *r*_*S,*_ = .456, *p* = .038, 19 df).

It is clear that the relationship of assessment time to postgraduate attainment is not due to confounding with entry qualifications. Do entry qualifications therefore have an independent prediction of postgraduate attainment? The partial correlation of postgraduate attainment with entry qualifications after taking assessment time into account was not significant (partial *r*_*S,*_ = .340, *p* = .131, 19 df). However, considering the simple correlations of assessment time and entry qualifications with postgraduate attainment (*r*_*S*_ = .515 and .447), the difference between these correlated correlations is not itself significant (*p* = .762 using the method of Meng *et al*. [29] although the N is small for such a calculation. Assessment time in these data therefore seems undoubtedly to be related to postgraduate attainment, but it is not clear whether entry qualifications are also related.

### Other measures

The UKFPO office publishes data on the mean performance of students from medical schools on its SJT and EPM. EPM, which it must be remembered is a measure of educational performance *within medical schools* did not correlate with postgraduate attainment (*r*_*S*_ = .224, *n* = 22, *p* = .316) or with assessment time (*r*_*S*_ = .210, *n* = 22, *p* = .349), and neither did SJT relate to postgraduate attainment (*r*_*S*_ = .046, *n* = 22, *p* = .316) or assessment time (*r*_*S*_ = −.225, *n* = 22, *p* = .313). However both EPM and SJT scores, which were somewhat correlated with each other (*r*_*S*_ = .374, *n* = 22, *p* = .086), correlated significantly with entry qualifications (*r*_*S*_ = .480 *n* = 22, *p* = .024; *r*_*S*_ = .493, *n* = 22, *p* = .020 respectively). Correlations with other measures are shown in Additional file 1.

Data were available on the mean levels of student satisfaction at each medical school, and it might be expected that students would be more satisfied at schools with less assessment, but the non-significant correlation was in fact positive (*r*_*S*_ = .134, *n* = 22, *p* = .553), with student satisfaction correlated with none of the other variables in the study (see Additional file 1). Relationships between other variables are presented in Additional file 1, but it should be remembered that with 23 non-independent variables there are 253 correlations reported, and therefore care should be taken in interpreting correlations to avoid Type 1 errors. Having said that, it is probable that, for instance, it makes sense for research intensity at medical schools to be correlated with longer courses and compulsory intercalated BScs (iBSC), with both then related to EPM, since intercalated degrees are a component of the EPM. Other researchers may find other relationships also to be of interest.

## Discussion

In implementing this study, we sought to gain a quantitative snapshot of assessment policy across the majority of medical schools in the UK. To our knowledge such data are not available anywhere else, and notably they are entirely absent from the GMC’s recent report entitled *How are students assessed at medical schools across the UK?* [30]*.* Whilst variance in undergraduate finals assessment has previously been evaluated by McCrorie *et al.* [3], previous analyses have been limited to qualitative data, which, whilst useful, offers less robust conclusions regarding the scale of variation. Our study goes further, documenting assessment in the most recent academic year for the entire undergraduate course at 25 of 27 A100 courses within the UK. We found substantial variation, not only in assessment volume, but also in the type and intensity of assessment. It was reassuring to us that the majority of schools we contacted were encouraging of our project and keen to learn how they compared with others. In discussing ‘assessment’ in the present paper we are almost entirely concerned with ‘summative assessment’ – formal examinations on which decisions are made about progression. Assessment is, of course, wider than that, and as Van der Vleuten *et al*. [31] have emphasized, there also needs to be ‘formative assessment’ or ‘assessment for learning’, in what should be a programme of assessment. In this study we have not attempted to collect data on formative assessment, and suspect it would be a harder task than collecting information about summative assessment. It would however be worthwhile, and future work needs to take it into account as well.

All UK medical students are assumed to meet a minimum competency standard as outlined by the GMC’s *Tomorrow’s Doctors* [32] The capacity of a school to provide a passing-level student with this competency is quality assured by the QABME inspection process. Given this, the schools with the lowest assessment volume presumably provide an estimate of the minimum acceptable volume of assessment. By that logic, all other schools in the UK are assessing students at a volume above and beyond that already deemed necessary for probing an appropriate level of medical knowledge, skills and attitudes. It is therefore reasonable to ask why some schools assess more than others and what the consequences may be. This we have attempted to do by correlating assessment volume with postgraduate examination outcomes. It should also be mentioned that there is a growing concern about what has been called “assessment overload” [33], although criticism acknowledges that “undoubtedly assessment is vital”.

The variation we report is considerable, with students at the University of Cambridge experiencing 3.2-fold more written assessment during their undergraduate careers than those at the University of Leeds. The scale of this variation is perhaps unsurprising given that medical schools develop assessment locally, without detailed knowledge of how other schools achieve similar goals, and without any central guidance. What was not clear to us was the potential impact of such variation on postgraduate performance.

### Assessment drives learning

Our exploratory analysis suggests, to use a frequent maxim from medical education, that ‘assessment drives learning’ [15], with students at assessment-heavy schools more likely to perform better in both knowledge assessments and clinical examinations of the MRCGP and MRCP(UK). That maxim from medical education, although rarely unpacked theoretically, is in fact strongly supported from a large number of psychological studies of the ‘testing effect’, in which retrieving information from memory, as in any form of testing, itself strengthens memories. Typical experimental studies involve initial learning, an intervening period which may or may not include a test, and a subsequent test of the material. A recent meta-analysis of 159 effect sizes from 61 studies found a mean effect size for the testing effect of 0.50 [34], and while the meta-analysis could not differentiate between several separate theoretical explanations for the effect, ‘effortful processing’ does seem to be important, with ‘depth of processing’ contributing in general to learning [35, 36]. Although studies are rare in medical education, two experimental studies with randomization do suggest that assessment does indeed improve learning [14, 37], with summative assessment in particular better than formative assessment [14]. It seems reasonable to conclude, therefore, that having a broader range of facts and skills prompted and reinforced through regular examination increases exposure to assessment and improves baseline knowledge. These findings provide a missing piece of the jigsaw that, in part, explains the variation in postgraduate assessment performance between medical schools [18]. Although our analysis on its own cannot directly assess the issue of causality, being correlational in nature, the existence of experimental, randomly controlled studies of the testing effect makes it at least plausible to infer that the variation we are describing has a causal influence on subsequent postgraduate outcomes. That would not be easy to test using a randomized controlled trial, but neither should it be impossible given sufficient will on the part of medical schools.

### Variation in entry qualifications

An intriguing aspect of the current data is that there is a positive, albeit non-significant, correlation of 0.303 between entry qualifications of medical students and the amount of assessment that they receive (Fig. 4). Although it could be argued that this correlation is non-significant and therefore should not be interpreted, the concept of significance is difficult when dealing with characteristics of institutions when those institutions are almost an entire sample of the institutions in the UK population of medical schools. It could be said that the correlation is therefore a descriptive statistic, and should not be tested for significance, which assumes random sampling from an infinite population. At the descriptive level, then, it can be asked why schools taking in more highly qualified entrants should assess *more* than those taking less qualified entrants. It might have been assumed that the more able students would need less testing, not more. There is therefore a possibility that assessment is acting as a ‘multiplier effect’, stretching even more the students who are already more able, and hence helping them to achieve even more. Certainly differences at postgraduate level are large (and for instance the first time scores at MRCP(UK) Part 1 [18], which correspond to pass rates from 91 % for Oxford graduates to 32 % for Liverpool graduates, seem potentially disproportionate to the relatively small differences in entry qualifications seen in our Fig. 4).

### EPM and SJT

The EPM cannot be expected to correlate at medical school level with postgraduate examinations or entry qualifications since it is primarily looking at variance *within* medical school rather than between. The EPM, which is scored out of 50, awards up to 43 points for medical school performance with the remainder allocated to additional degrees (five points) and publications (two points) [38]. The fact that EPM does correlate with MRCP(UK) Parts 1 and 2 (see Additional files 1 and 2) probably reflects the fact that intercalated degrees are included within the EPM, and all students at some medical schools take those degrees, and those schools are also those with higher entry qualifications, with which EPM also correlates. Schools with compulsory intercalated degrees have higher scores on the EPM than do other schools, as also do schools with 6 year courses (see Additional files 1 and 2), and are also more research intensive. Students attending these same research intensive schools may be more likely to publish either as a result of compulsory intercalated degrees or because of an ethos of active participation in research activities. The UKFPO SJT has been said to be the closest that there is in the UK to a national licensing examination, the same examination being sat by all students at all UK medical schools, with large differences in mean scores being apparent. The SJT is said to be an assessment which “cannot be revised for, but [for which] you can prepare” [39] and is explicitly an assessment of “aptitude” rather than clinical knowledge [40]. Consistent with that, SJT scores across medical schools do not correlate with postgraduate attainment, although scores are higher in schools with higher entry qualifications (see Additional files 1 and 2). The correlation of overall assessment time with postgraduate outcomes but not with SJTs, suggests that assessment time is not merely about ‘test-wiseness’ but rather is about encouraging a greater amount of medical knowledge which is beneficial when taking postgraduate assessments.

### The stability of measures

The data for the present correlational studies are complex in that they are snapshots taken at different moments in time, sometimes averaged over several years. It is also the case that postgraduate examinations are taken several or more years after leaving medical school, and other educational training has taken place during that time. MRCP(UK) results are for 2002–2013, whereas entry standards are for 2012 entry, and assessment times were collected in 2014 but apply to all 5 or 6 years of the medical school course. Of necessity we have therefore correlated institution level data from snapshots which are separated in time. In an ideal world there would be detailed longitudinal data across a decade or more of university entrants, following their careers over the next decade or more as they progress through medical school and into postgraduate training and examinations. That paragon of perfection does not exist; and it seems unlikely to be straightforward to obtain it retrospectively. A key assumption for the present analyses to be valid is that there is reasonable stability of institutions across time. Given the absence of comprehensive record keeping by individual institutions over time, we examined the only previously published comparative data on medical school assessments by McCrorie *et al*. [3] (and we thank the authors for providing us with the raw data for that study). We correlated the total volume (in minutes) of written and practical final year assessment at each institution (collected in 2006) with our own data (collected in 2014). We found a strong, statistically significant correlation (*r* = .703, *p* = .0003; r_s_ = .694, *p* = .0003) between finals assessment practices, supporting our earlier assumptions regarding the stability of assessment practices over time (data not shown). In the case of MRCP(UK) we have shown for the Part 1 assessment that mean medical school scores correlate .785 across 1 year, .689 across 5 years, .669 across 10 years, and .634 across 15 years, suggesting that there is good stability of medical school differences over time. Data for entry tariffs are harder to obtain since units of measurements change but the nominal ‘2015’ values we quote above (actually based on 2012 entrants) correlate .899 with ‘2013’ (i.e. 2010 entrant) data, .799 with ‘2010’ (i.e. 2007 entrant) data, and .747 with ‘2008’ (i.e. 2005 entrant) data, the latter all taken from university league tables summarized by and published in *The Guardian*, a UK national newspaper. Once again there is the suggestion of a large degree of stability in the measures. However some of the variation between years necessarily reflects random measurement error, and hence the true, disattenuated correlations are probably higher.

It should also be said that if measures are unreliable due to instability or due to measurement error, then such lack of reliability (stability) will necessarily reduce measured correlations between variables. Considering, say, the correlation between volume of assessment and postgraduate examination performance of r_s_ = .515 (see Results section), for which the reliability across time of volume of assessment is r_s_ = .694 (see previous paragraph), and the reliability of measurement of postgraduate exams is of the order of .689 (the 5 year figure for MRCP(UK) in the previous paragraph). Using the conventional formula for disattenuating a correlation for unreliability, then the true disattenuated correlation is .515/sqrt(.694 × .689) = .744. The true correlation of .744 suggests that about 55 % of the true variation in postgraduate performance is a function of different assessment volume at medical school. Many of the correlations reported here are therefore likely to be conservative estimates of the true correlations.

### The design of future studies

The previous paragraphs have suggested that there potentially are statistical problems with any comparison of institutional policies which relies on correlations of aggregated means (and for that reason we regard the present study as exploratory), with the main difficulty being that the number of institutions is small for assessing statistical significance. A potential solution to that might be found by considering the scatterplot in Fig. 4 which shows total minutes of assessment in the medical schools in relation to entry qualifications. Consider the medical schools, which have broadly similar entry qualifications but a wide range of assessment times. A study of individuals at three medical schools at the top of the box (Queen’s University Belfast; University of Newcastle; University of Bristol) and three at the bottom of the box (University of Leeds; University of Sheffield; and University of Manchester or St George’s (University of London)) allows a strong *a priori* prediction that on a comparable outcome measure (say, a Royal College examination, or perhaps MCQ items generated by the MSC-AA, or, in the future, a national licensing examination) students from schools at the top of the box should perform better than those at the bottom of the box, even taking individual entry qualifications into account. Statistical analysis would be by multi-level modelling, which would give more power, students being clustered within schools.

### Implications for a national licensing examination

Numerous stakeholders (including the GMC) have expressed concerns that a system of standardised national examination in the UK might not achieve the same volume or range of assessment as currently exists throughout a standard UK medical degree. Although international comparative data are scarce, a useful source of data is the USMLE assessment, in which US medical students sit three examinations at intervals throughout their early medical training. Passing the USMLE is an essential requirement for independent medical practice in the United States. The USMLE sits comfortably within the range of our data set, with a total of 1680 min of assessment, comprising 1157 MCQ items taken over 1500 min, giving a mean time per item of 1.30 min. Interestingly, the prescribed volume of assessment of the USMLE is greater than or equal to the total volumes of written assessment at five UK medical schools. It is important to note though that the USMLE is far from the only assessment taken by US undergraduate students, with US medical schools also setting local assessments in addition to the USMLE, and so most US students will have taken many more assessments than USMLE alone. As of June 2015, no announcements had been made about the likely format, length and intensity of the assessments likely to be included in the new UKMLA.

## Conclusions

This study has quantified and compared previously undocumented details of the undergraduate assessment experience at UK medical schools. We have demonstrated substantial variation in the volume, type and intensity of undergraduate assessment. Furthermore, our data, taken alongside those of the USMLE, allay frequently cited concerns regarding the length, frequency and intensity of any potential system of national assessment. In the context of the variation documented here, a stepwise system has the potential to offer a robust solution to standardised assessment, clearly defined progression policies and fair ranking of students for entry into the Foundation Programme. Taken together, our findings suggest that a closer examination of the implications of locally variable assessment policy is warranted.

## Additional files

Additional file 1: **Correlation matrix for all variables in the study.** (DOCX 23 kb) Additional file 2: **Correlations of Additional File 1 as an Excel Spreadsheet.** (DOCX 61 kb)

## Abbreviations

A100: Primary UCAS code for medical courses
AKT: Applied Knowledge Test
BMA-MSC: British Medical Association’s Medical Student Committee
CSA: Clinical Skills Assessment
EMQ: Extended Matching Question
EPM: Educational Performance Measure
FPAS: Foundation Programme Application System
GMC: General Medical Council
HESA: Higher Education Statistics Agency
IBM SPSS: IBM Statistical Package for the Social Sciences
iBSc: Intercalated or Integrated Bachelor of Science
LQ: Lower quartile
MCQ: Multiple Choice Question
MRCGP: Membership of the Royal College of General Practitioners
MRCP(UK): Membership of the Royal Colleges of Physicians of the United Kingdom
MSC-AA: Medical Schools’ Council Assessment Alliance
NSS: National Student Survey
OSCE: Objective Structured Clinical Examination
PACES: Practical Assessment of Clinical and Examination Skills
QAA: Quality Assurance Agency for Higher Education
QABME: Quality Assurance of Basic Medical Education
RAE: Research Assessment Exercise
SAQ: Short Answer Question
SBA: Single Best Answer
SD: Standard deviation
SJT: Situational Judgement Test
UCAS: Universities and Colleges Admissions Service
UK: United Kingdom
UKFPO: United Kingdom Foundation Programme Office
UKMLA: United Kingdom Medical Licensing Assessment
UQ: Upper quartile
USMLE: United States Medical Licensing Examination
