# Supplementary material for: Assessment at UK medical schools varies substantially in volume, type and intensity and correlates with postgraduate attainment
Source: BMC Med Educ. 2015 Sep 11;15:146. doi: 10.1186/s12909-015-0428-9 (PMC4567823; doi:10.1186/s12909-015-0428-9)
Supplement: Additional file 1: — Correlation matrix for all variables in the study. (DOCX 23 kb) [file 12909_2015_428_MOESM1_ESM.docx]

**Table 1** Volume of written and practical assessment at UK medical schools. Data are divided into preclinical (years 1 and 2) and clinical (years 3, 4 and 5). Minutes and marks are rounded to the nearest hundred.

|  | |  | | | | **Summative, Timed, Written Assessment** | | | | | | | | **Practical Assessment** | | |
| --- | --- | --- | --- | --- | --- | --- | --- | --- | --- | --- | --- | --- | --- | --- | --- | --- |
|  | |  |  |  |  | ***Minutes*** | | | | ***Marks*** | | | ***Intensity*** | ***Minutes*** | | |
|  | **School** | | **UCAS Code** | **Course Length** | **Compulsory Intercalation** | **Pre-clinical** | **Clinical** | **Total** | **Pre-clinical** | | **Clinical** | **Total** | **min/ mark** | **Pre-clinical** | **Clinical** | **Total** |
| 1 | Brighton & Sussex Medical School | | B74 | 5 | N | 1100 | 1100 | 2200 | 400 | | 800 | 1200 | 1.9 | 100 | 300 | 400 |
| 2 | Cardiff University | | C15 | 5 | N | 900 | 1100 | 2000 | 700 | | 800 | 1500 | 1.36 | 0 | 200 | 200 |
| 3 | Hull and York Medical School | | H75 | 5 | N | 700 | 800 | 1500 | 800 | | 500 | 1300 | 1.13 | 300 | 700 | 1000 |
| 4 | Imperial College London | | I50 | 6 | Y | 900 | 1000 | 1900 | 1100 | | 1000 | 2100 | 0.95 | 0 | 400 | 400 |
| 5 | Keele University | | K12 | 5 | N | 700 | 600 | 1300 | 600 | | 500 | 1100 | 1.20 | 300 | 400 | 700 |
| 6 | King’s College London | | K60 | 5 | N | 1100 | 1000 | 2100 | 900 | | 600 | 1500 | 1.38 | 100 | 400 | 500 |
| 7 | Newcastle University | | N21 | 5 | N | 1600 | 1100 | 2700 | 1300 | | 800 | 2100 | 1.23 | 100 | 200 | 300 |
| 8 | Queen Mary, University of London | | Q50 | 5 | N | 1800 | 1000 | 2800 | 1900 | | 900 | 2800 | 1.02 | 100 | 500 | 600 |
| 9 | Queen’s University Belfast | | Q75 | 5 | N | 1700 | 1100 | 2800 | ? | | ? | ? | ? | 100 | 300 | 400 |
| 10 | St George’s, University of London | | S49 | 5 | N | 700 | 1100 | 1800 | 500 | | 800 | 1300 | 1.40 | 100 | 500 | 600 |
| 11 | The University of Edinburgh | | E56 | 5 | N | 1100 | 1300 | 2400 | ? | | ? | ? | ? | 100 | 200 | 300 |
| 12 | The University of Sheffield | | S18 | 5 | N | 700 | 800 | 1500 | 700 | | 800 | 1500 | 1.04 | 300 | 100 | 400 |
| 13 | University College London | | U80 | 6 | Y | 800 | 900 | 1700 | 600 | | 800 | 1400 | 1.29 | 0 | 400 | 400 |
| 14 | University of Aberdeen | | A20 | 5 | N | 600 | 900 | 1500 | 500 | | 800 | 1300 | 1.15 | 100 | 300 | 400 |
| 15 | University of Birmingham | | B32 | 5 | N | 1600 | 1200 | 2800 | 1500 | | 800 | 2300 | 1.24 | 0 | 300 | 300 |
| 16 | University of Bristol | | B78 | 5 | N | 1200 | 1200 | 2400 | ? | | ? | ? | ? | ? | ? | ? |
| 17 | University of Cambridge | | C05 | 6 | Y | 2300 | 900 | 3200 | 900 | | 600 | 1500 | 2.08 | 0 | 800 | 800 |
| 18 | University of Dundee | | D65 | 5 | N | 800 | 700 | 1500 | ? | | ? | ? | ? | 200 | 400 | 600 |
| 19 | University of Glasgow | | G28 | 5 | N | 800 | 800 | 1600 | 500 | | 700 | 1200 | 1.25 | 100 | 300 | 400 |
| 20 | University of Leeds | | L23 | 5 | N | 500 | 500 | 1000 | 600 | | 600 | 1200 | 0.85 | 0 | 400 | 400 |
| 21 | University of Liverpool | | L41 | 5 | N | 600 | 600 | 1200 | 600 | | 600 | 1200 | 0.97 | 400 | 500 | 900 |
| 22 | University of Manchester | | M20 | 5 | N | 1200 | 600 | 1800 | 1000 | | 500 | 1500 | 1.20 | 100 | 300 | 400 |
| 23 | University of Nottingham | | N84 | 5 | Y | 1500 | 900 | 2400 | ? | | ? | ? | ? | ? | ? | ? |
| 24 | University of Oxford | | O33 | 6 | Y | 1600 | 1100 | 2700 | 900 | | 1100 | 2000 | 1.37 | 0 | 500 | 500 |
| 25 | University of Southampton | | S27 | 5 | Y | 1000 | 900 | 1900 | 600 | | 600 | 1200 | 1.52 | 0 | 200 | 200 |

*^?^ - Indicates data that schools were unwilling or unable to validate or provide.*
