# Supplementary material for: Assessment at UK medical schools varies substantially in volume, type and intensity and correlates with postgraduate attainment
Source: BMC Med Educ. 2015 Sep 11;15:146. doi: 10.1186/s12909-015-0428-9 (PMC4567823; doi:10.1186/s12909-015-0428-9)
Supplement: Additional file 2: — Correlations of Additional File 1 as an Excel Spreadsheet. (DOCX 61 kb) [file 12909_2015_428_MOESM2_ESM.docx]

Additional File 1

**Correlations between all variables**.

The table below shows a complete correlation matrix for the 22 medical schools for which adequate data was available for postgraduate assessments (i.e. University of Aberdeen, Queen Mary (University of London), University of Birmingham, University of Bristol, University of Cambridge, Cardiff University, University of Dundee, The University of Edinburgh, University of Glasgow, Imperial College London, King's College London, University of Leeds, University of Liverpool, University of Manchester, Newcastle University, University of Nottingham, University of Oxford, Queen's University Belfast, St George's (University of London), The University of Sheffield, University of Southampton, and University College London).

Correlations are Spearman, rank-order correlations which are non-parametric.

**Note**: The table on the next page uses a large and irregular paper size in order that the table will fit onto a single page. It may not be possible, therefore, to print the table, and it is best looked at on screen. The table is also available as an Excel spreadsheet in Additional File 2.

| **Spearman correlations** |  |  |  |  |  |  |  |  |  |  |  |  |  |  |  |  |  |  |  |  |  |  |  |  |  |  |
| --- | --- | --- | --- | --- | --- | --- | --- | --- | --- | --- | --- | --- | --- | --- | --- | --- | --- | --- | --- | --- | --- | --- | --- | --- | --- | --- |
|  | | Minutes Total | Minutes Preclinical | Minutes Clinical | Marks Total | Marks Preclinical | Marks Clinical | Assess't volume | Practical Total | Practical Preclinical | Practical Clinical | Mean PG attain't | MRCGP AKT | MRCGP CSA | MRCP Part 1 | MRCP Part 2 | MRCP PACES | UKFPO EPM | UKFPO SJT | Entry Tariff | Student Satisf'n | Research grading | Course Length | Comp'y IBSc |  |  |
| Minutes Total | Correlation Coefficient | **1.000** | **.951^**^** | **.703^**^** | **.752^**^** | **.726^**^** | **.287** | **.478** | **-.108** | **-.361** | **.075** | **.515^*^** | **.598^**^** | **.514^*^** | **.646^**^** | **.610^**^** | **.589^*^** | **.210** | **-.229** | **.303** | **.134** | **.306** | **.205** | **.218** |  |  |
|  | Sig. (2-tailed) |  | **.000** | **.000** | **.001** | **.001** | **.265** | **.052** | **.650** | **.117** | **.753** | **.014** | **.003** | **.014** | **.005** | **.009** | **.013** | **.349** | **.305** | **.170** | **.553** | **.166** | **.360** | **.330** |  |  |
|  | N | **22** | **22** | **22** | **17** | **17** | **17** | **17** | **20** | **20** | **20** | **22** | **22** | **22** | **17** | **17** | **17** | **22** | **22** | **22** | **22** | **22** | **22** | **22** |  |  |
| Minutes Preclinical | Correlation Coefficient | **.951^**^** | **1.000** | **.508^*^** | **.732^**^** | **.781^**^** | **.155** | **.401** | **-.049** | **-.263** | **.073** | **.424^*^** | **.500^*^** | **.418** | **.591^*^** | **.550^*^** | **.546^*^** | **.222** | **-.101** | **.301** | **.227** | **.329** | **.196** | **.234** |  |  |
|  | Sig. (2-tailed) | **.000** |  | **.016** | **.001** | **.000** | **.552** | **.110** | **.838** | **.262** | **.761** | **.049** | **.018** | **.053** | **.013** | **.022** | **.023** | **.320** | **.653** | **.173** | **.310** | **.134** | **.383** | **.294** |  |  |
|  | N | **22** | **22** | **22** | **17** | **17** | **17** | **17** | **20** | **20** | **20** | **22** | **22** | **22** | **17** | **17** | **17** | **22** | **22** | **22** | **22** | **22** | **22** | **22** |  |  |
| Minutes Clinical | Correlation Coefficient | **.703^**^** | **.508^*^** | **1.000** | **.642^**^** | **.417** | **.634^**^** | **.400** | **-.350** | **-.274** | **-.163** | **.497^*^** | **.572^**^** | **.489^*^** | **.576^*^** | **.511^*^** | **.492^*^** | **.025** | **-.450^*^** | **.115** | **-.080** | **.142** | **.038** | **-.049** |  |  |
|  | Sig. (2-tailed) | **.000** | **.016** |  | **.005** | **.096** | **.006** | **.112** | **.130** | **.243** | **.492** | **.019** | **.005** | **.021** | **.016** | **.036** | **.045** | **.913** | **.036** | **.610** | **.722** | **.528** | **.868** | **.829** |  |  |
|  | N | **22** | **22** | **22** | **17** | **17** | **17** | **17** | **20** | **20** | **20** | **22** | **22** | **22** | **17** | **17** | **17** | **22** | **22** | **22** | **22** | **22** | **22** | **22** |  |  |
| Marks Total | Correlation Coefficient | **.752^**^** | **.732^**^** | **.642^**^** | **1.000** | **.906^**^** | **.583^*^** | **-.084** | **-.050** | **-.150** | **.033** | **.276** | **.258** | **.258** | **.613^*^** | **.540** | **.573** | **.251** | **-.206** | **.420** | **.216** | **.406** | **.245** | **.054** |  |  |
|  | Sig. (2-tailed) | **.001** | **.001** | **.005** |  | **.000** | **.014** | **.749** | **.849** | **.564** | **.899** | **.283** | **.318** | **.318** | **.034** | **.070** | **.052** | **.331** | **.427** | **.093** | **.404** | **.106** | **.342** | **.838** |  |  |
|  | N | **17** | **17** | **17** | **17** | **17** | **17** | **17** | **17** | **17** | **17** | **17** | **17** | **17** | **12** | **12** | **12** | **17** | **17** | **17** | **17** | **17** | **17** | **17** |  |  |
| Marks Preclinical | Correlation Coefficient | **.726^**^** | **.781^**^** | **.417** | **.906^**^** | **1.000** | **.267** | **-.161** | **-.061** | **-.155** | **.032** | **.192** | **.205** | **.177** | **.577^*^** | **.535** | **.450** | **.215** | **-.053** | **.267** | **.167** | **.412** | **.158** | **.053** |  |  |
|  | Sig. (2-tailed) | **.001** | **.000** | **.096** | **.000** |  | **.300** | **.537** | **.817** | **.552** | **.902** | **.460** | **.431** | **.496** | **.049** | **.073** | **.143** | **.408** | **.839** | **.299** | **.522** | **.100** | **.546** | **.839** |  |  |
|  | N | **17** | **17** | **17** | **17** | **17** | **17** | **17** | **17** | **17** | **17** | **17** | **17** | **17** | **12** | **12** | **12** | **17** | **17** | **17** | **17** | **17** | **17** | **17** |  |  |
| Marks Clinical | Correlation Coefficient | **.287** | **.155** | **.634^**^** | **.583^*^** | **.267** | **1.000** | **-.147** | **-.048** | **-.175** | **.063** | **.273** | **.226** | **.232** | **.340** | **.299** | **.477** | **.224** | **-.299** | **.483^*^** | **.400** | **.207** | **.357** | **.194** |  |  |
|  | Sig. (2-tailed) | **.265** | **.552** | **.006** | **.014** | **.300** |  | **.574** | **.854** | **.501** | **.810** | **.289** | **.382** | **.371** | **.280** | **.345** | **.117** | **.388** | **.244** | **.050** | **.111** | **.424** | **.160** | **.456** |  |  |
|  | N | **17** | **17** | **17** | **17** | **17** | **17** | **17** | **17** | **17** | **17** | **17** | **17** | **17** | **12** | **12** | **12** | **17** | **17** | **17** | **17** | **17** | **17** | **17** |  |  |
| Assessment volume | Correlation Coefficient | **.478** | **.401** | **.400** | **-.084** | **-.161** | **-.147** | **1.000** | **-.009** | **-.306** | **.086** | **.358** | **.370** | **.321** | **.769^**^** | **.566** | **.420** | **.324** | **.020** | **.070** | **-.281** | **.117** | **.198** | **.369** |  |  |
|  | Sig. (2-tailed) | **.052** | **.110** | **.112** | **.749** | **.537** | **.574** |  | **.973** | **.233** | **.742** | **.158** | **.144** | **.209** | **.003** | **.055** | **.175** | **.205** | **.940** | **.790** | **.275** | **.656** | **.446** | **.145** |  |  |
|  | N | **17** | **17** | **17** | **17** | **17** | **17** | **17** | **17** | **17** | **17** | **17** | **17** | **17** | **12** | **12** | **12** | **17** | **17** | **17** | **17** | **17** | **17** | **17** |  |  |
| Practical Total | Correlation Coefficient | **-.108** | **-.049** | **-.350** | **-.050** | **-.061** | **-.048** | **-.009** | **1.000** | **.374** | **.858^**^** | **-.370** | **-.393** | **-.330** | **-.248** | **-.230** | **-.115** | **.364** | **.266** | **.032** | **-.129** | **.064** | **.236** | **.031** |  |  |
|  | Sig. (2-tailed) | **.650** | **.838** | **.130** | **.849** | **.817** | **.854** | **.973** |  | **.104** | **.000** | **.108** | **.086** | **.155** | **.372** | **.410** | **.683** | **.114** | **.257** | **.895** | **.588** | **.789** | **.316** | **.896** |  |  |
|  | N | **20** | **20** | **20** | **17** | **17** | **17** | **17** | **20** | **20** | **20** | **20** | **20** | **20** | **15** | **15** | **15** | **20** | **20** | **20** | **20** | **20** | **20** | **20** |  |  |
| Practical Preclinical | Correlation Coefficient | **-.361** | **-.263** | **-.274** | **-.150** | **-.155** | **-.175** | **-.306** | **.374** | **1.000** | **-.099** | **-.586^**^** | **-.653^**^** | **-.502^*^** | **-.619^*^** | **-.691^**^** | **-.269** | **-.490^*^** | **-.096** | **-.453^*^** | **-.060** | **-.388** | **-.565^**^** | **-.653^**^** |  |  |
|  | Sig. (2-tailed) | **.117** | **.262** | **.243** | **.564** | **.552** | **.501** | **.233** | **.104** |  | **.678** | **.007** | **.002** | **.024** | **.014** | **.004** | **.332** | **.028** | **.688** | **.045** | **.803** | **.091** | **.009** | **.002** |  |  |
|  | N | **20** | **20** | **20** | **17** | **17** | **17** | **17** | **20** | **20** | **20** | **20** | **20** | **20** | **15** | **15** | **15** | **20** | **20** | **20** | **20** | **20** | **20** | **20** |  |  |
| Practical Clinical | Correlation Coefficient | **.075** | **.073** | **-.163** | **.033** | **.032** | **.063** | **.086** | **.858^**^** | **-.099** | **1.000** | **-.124** | **-.109** | **-.139** | **.006** | **.064** | **-.057** | **.634^**^** | **.299** | **.236** | **-.124** | **.254** | **.477^*^** | **.297** |  |  |
|  | Sig. (2-tailed) | **.753** | **.761** | **.492** | **.899** | **.902** | **.810** | **.742** | **.000** | **.678** |  | **.603** | **.647** | **.558** | **.984** | **.820** | **.840** | **.003** | **.201** | **.317** | **.602** | **.280** | **.033** | **.203** |  |  |
|  | N | **20** | **20** | **20** | **17** | **17** | **17** | **17** | **20** | **20** | **20** | **20** | **20** | **20** | **15** | **15** | **15** | **20** | **20** | **20** | **20** | **20** | **20** | **20** |  |  |
| Mean PG attainment | Correlation Coefficient | **.515^*^** | **.424^*^** | **.497^*^** | **.276** | **.192** | **.273** | **.358** | **-.370** | **-.586^**^** | **-.124** | **1.000** | **.930^**^** | **.946^**^** | **.924^**^** | **.953^**^** | **.873^**^** | **.224** | **.047** | **.447^*^** | **.019** | **.147** | **.390** | **.402** |  |  |
|  | Sig. (2-tailed) | **.014** | **.049** | **.019** | **.283** | **.460** | **.289** | **.158** | **.108** | **.007** | **.603** |  | **.000** | **.000** | **.000** | **.000** | **.000** | **.316** | **.836** | **.037** | **.932** | **.514** | **.073** | **.064** |  |  |
|  | N | **22** | **22** | **22** | **17** | **17** | **17** | **17** | **20** | **20** | **20** | **22** | **22** | **22** | **17** | **17** | **17** | **22** | **22** | **22** | **22** | **22** | **22** | **22** |  |  |
| MRCGP AKT | Correlation Coefficient | **.598^**^** | **.500^*^** | **.572^**^** | **.258** | **.205** | **.226** | **.370** | **-.393** | **-.653^**^** | **-.109** | **.930^**^** | **1.000** | **.896^**^** | **.816^**^** | **.848^**^** | **.721^**^** | **.197** | **-.010** | **.387** | **.059** | **.201** | **.390** | **.402** |  |  |
|  | Sig. (2-tailed) | **.003** | **.018** | **.005** | **.318** | **.431** | **.382** | **.144** | **.086** | **.002** | **.647** | **.000** |  | **.000** | **.000** | **.000** | **.001** | **.379** | **.966** | **.075** | **.795** | **.369** | **.073** | **.064** |  |  |
|  | N | **22** | **22** | **22** | **17** | **17** | **17** | **17** | **20** | **20** | **20** | **22** | **22** | **22** | **17** | **17** | **17** | **22** | **22** | **22** | **22** | **22** | **22** | **22** |  |  |
| MRCGP CSA | Correlation Coefficient | **.514^*^** | **.418** | **.489^*^** | **.258** | **.177** | **.232** | **.321** | **-.330** | **-.502^*^** | **-.139** | **.946^**^** | **.896^**^** | **1.000** | **.860^**^** | **.926^**^** | **.811^**^** | **.158** | **-.054** | **.278** | **.112** | **.044** | **.297** | **.322** |  |  |
|  | Sig. (2-tailed) | **.014** | **.053** | **.021** | **.318** | **.496** | **.371** | **.209** | **.155** | **.024** | **.558** | **.000** | **.000** |  | **.000** | **.000** | **.000** | **.484** | **.813** | **.211** | **.620** | **.848** | **.179** | **.144** |  |  |
|  | N | **22** | **22** | **22** | **17** | **17** | **17** | **17** | **20** | **20** | **20** | **22** | **22** | **22** | **17** | **17** | **17** | **22** | **22** | **22** | **22** | **22** | **22** | **22** |  |  |
| MRCP Part 1 | Correlation Coefficient | **.646^**^** | **.591^*^** | **.576^*^** | **.613^*^** | **.577^*^** | **.340** | **.769^**^** | **-.248** | **-.619^*^** | **.006** | **.924^**^** | **.816^**^** | **.860^**^** | **1.000** | **.958^**^** | **.792^**^** | **.600^*^** | **.154** | **.587^*^** | **-.145** | **.495^*^** | **.559^*^** | **.510^*^** |  |  |
|  | Sig. (2-tailed) | **.005** | **.013** | **.016** | **.034** | **.049** | **.280** | **.003** | **.372** | **.014** | **.984** | **.000** | **.000** | **.000** |  | **.000** | **.000** | **.011** | **.554** | **.013** | **.579** | **.043** | **.020** | **.037** |  |  |
|  | N | **17** | **17** | **17** | **12** | **12** | **12** | **12** | **15** | **15** | **15** | **17** | **17** | **17** | **17** | **17** | **17** | **17** | **17** | **17** | **17** | **17** | **17** | **17** |  |  |
| MRCP Part 2 | Correlation Coefficient | **.610^**^** | **.550^*^** | **.511^*^** | **.540** | **.535** | **.299** | **.566** | **-.230** | **-.691^**^** | **.064** | **.953^**^** | **.848^**^** | **.926^**^** | **.958^**^** | **1.000** | **.806^**^** | **.635^**^** | **.152** | **.600^*^** | **-.010** | **.446** | **.559^*^** | **.538^*^** |  |  |
|  | Sig. (2-tailed) | **.009** | **.022** | **.036** | **.070** | **.073** | **.345** | **.055** | **.410** | **.004** | **.820** | **.000** | **.000** | **.000** | **.000** |  | **.000** | **.006** | **.560** | **.011** | **.970** | **.073** | **.020** | **.026** |  |  |
|  | N | **17** | **17** | **17** | **12** | **12** | **12** | **12** | **15** | **15** | **15** | **17** | **17** | **17** | **17** | **17** | **17** | **17** | **17** | **17** | **17** | **17** | **17** | **17** |  |  |
| MRCP PACES | Correlation Coefficient | **.589^*^** | **.546^*^** | **.492^*^** | **.573** | **.450** | **.477** | **.420** | **-.115** | **-.269** | **-.057** | **.873^**^** | **.721^**^** | **.811^**^** | **.792^**^** | **.806^**^** | **1.000** | **.238** | **.125** | **.541^*^** | **.048** | **.169** | **.410** | **.311** |  |  |
|  | Sig. (2-tailed) | **.013** | **.023** | **.045** | **.052** | **.143** | **.117** | **.175** | **.683** | **.332** | **.840** | **.000** | **.001** | **.000** | **.000** | **.000** |  | **.358** | **.633** | **.025** | **.855** | **.516** | **.102** | **.224** |  |  |
|  | N | **17** | **17** | **17** | **12** | **12** | **12** | **12** | **15** | **15** | **15** | **17** | **17** | **17** | **17** | **17** | **17** | **17** | **17** | **17** | **17** | **17** | **17** | **17** |  |  |
| UKFPO EPM | Correlation Coefficient | **.210** | **.222** | **.025** | **.251** | **.215** | **.224** | **.324** | **.364** | **-.490^*^** | **.634^**^** | **.224** | **.197** | **.158** | **.600^*^** | **.635^**^** | **.238** | **1.000** | **.369** | **.480^*^** | **-.033** | **.521^*^** | **.669^**^** | **.643^**^** |  |  |
|  | Sig. (2-tailed) | **.349** | **.320** | **.913** | **.331** | **.408** | **.388** | **.205** | **.114** | **.028** | **.003** | **.316** | **.379** | **.484** | **.011** | **.006** | **.358** |  | **.091** | **.024** | **.883** | **.013** | **.001** | **.001** |  |  |
|  | N | **22** | **22** | **22** | **17** | **17** | **17** | **17** | **20** | **20** | **20** | **22** | **22** | **22** | **17** | **17** | **17** | **22** | **22** | **22** | **22** | **22** | **22** | **22** |  |  |
| UKFPO SJT | Correlation Coefficient | **-.229** | **-.101** | **-.450^*^** | **-.206** | **-.053** | **-.299** | **.020** | **.266** | **-.096** | **.299** | **.047** | **-.010** | **-.054** | **.154** | **.152** | **.125** | **.369** | **1.000** | **.495^*^** | **-.155** | **.394** | **.483^*^** | **.386** |  |  |
|  | Sig. (2-tailed) | **.305** | **.653** | **.036** | **.427** | **.839** | **.244** | **.940** | **.257** | **.688** | **.201** | **.836** | **.966** | **.813** | **.554** | **.560** | **.633** | **.091** |  | **.019** | **.491** | **.069** | **.023** | **.076** |  |  |
|  | N | **22** | **22** | **22** | **17** | **17** | **17** | **17** | **20** | **20** | **20** | **22** | **22** | **22** | **17** | **17** | **17** | **22** | **22** | **22** | **22** | **22** | **22** | **22** |  |  |
| Entry Tariff | Correlation Coefficient | **.303** | **.301** | **.115** | **.420** | **.267** | **.483^*^** | **.070** | **.032** | **-.453^*^** | **.236** | **.447^*^** | **.387** | **.278** | **.587^*^** | **.600^*^** | **.541^*^** | **.480^*^** | **.495^*^** | **1.000** | **.199** | **.580^**^** | **.650^**^** | **.434^*^** |  |  |
|  | Sig. (2-tailed) | **.170** | **.173** | **.610** | **.093** | **.299** | **.050** | **.790** | **.895** | **.045** | **.317** | **.037** | **.075** | **.211** | **.013** | **.011** | **.025** | **.024** | **.019** |  | **.375** | **.005** | **.001** | **.043** |  |  |
|  | N | **22** | **22** | **22** | **17** | **17** | **17** | **17** | **20** | **20** | **20** | **22** | **22** | **22** | **17** | **17** | **17** | **22** | **22** | **22** | **22** | **22** | **22** | **22** |  |  |
| Student Satisaction | Correlation Coefficient | **.134** | **.227** | **-.080** | **.216** | **.167** | **.400** | **-.281** | **-.129** | **-.060** | **-.124** | **.019** | **.059** | **.112** | **-.145** | **-.010** | **.048** | **-.033** | **-.155** | **.199** | **1.000** | **-.011** | **-.028** | **.056** |  |  |
|  | Sig. (2-tailed) | **.553** | **.310** | **.722** | **.404** | **.522** | **.111** | **.275** | **.588** | **.803** | **.602** | **.932** | **.795** | **.620** | **.579** | **.970** | **.855** | **.883** | **.491** | **.375** |  | **.962** | **.902** | **.803** |  |  |
|  | N | **22** | **22** | **22** | **17** | **17** | **17** | **17** | **20** | **20** | **20** | **22** | **22** | **22** | **17** | **17** | **17** | **22** | **22** | **22** | **22** | **22** | **22** | **22** |  |  |
| Research grading | Correlation Coefficient | **.306** | **.329** | **.142** | **.406** | **.412** | **.207** | **.117** | **.064** | **-.388** | **.254** | **.147** | **.201** | **.044** | **.495^*^** | **.446** | **.169** | **.521^*^** | **.394** | **.580^**^** | **-.011** | **1.000** | **.595^**^** | **.378** |  |  |
|  | Sig. (2-tailed) | **.166** | **.134** | **.528** | **.106** | **.100** | **.424** | **.656** | **.789** | **.091** | **.280** | **.514** | **.369** | **.848** | **.043** | **.073** | **.516** | **.013** | **.069** | **.005** | **.962** |  | **.004** | **.083** |  |  |
|  | N | **22** | **22** | **22** | **17** | **17** | **17** | **17** | **20** | **20** | **20** | **22** | **22** | **22** | **17** | **17** | **17** | **22** | **22** | **22** | **22** | **22** | **22** | **22** |  |  |
| Course Length | Correlation Coefficient | **.205** | **.196** | **.038** | **.245** | **.158** | **.357** | **.198** | **.236** | **-.565^**^** | **.477^*^** | **.390** | **.390** | **.297** | **.559^*^** | **.559^*^** | **.410** | **.669^**^** | **.483^*^** | **.650^**^** | **-.028** | **.595^**^** | **1.000** | **.770^**^** |  |  |
|  | Sig. (2-tailed) | **.360** | **.383** | **.868** | **.342** | **.546** | **.160** | **.446** | **.316** | **.009** | **.033** | **.073** | **.073** | **.179** | **.020** | **.020** | **.102** | **.001** | **.023** | **.001** | **.902** | **.004** |  | **.000** |  |  |
|  | N | **22** | **22** | **22** | **17** | **17** | **17** | **17** | **20** | **20** | **20** | **22** | **22** | **22** | **17** | **17** | **17** | **22** | **22** | **22** | **22** | **22** | **22** | **22** |  |  |
| Compulsory IBSc | Correlation Coefficient | **.218** | **.234** | **-.049** | **.054** | **.053** | **.194** | **.369** | **.031** | **-.653^**^** | **.297** | **.402** | **.402** | **.322** | **.510^*^** | **.538^*^** | **.311** | **.643^**^** | **.386** | **.434^*^** | **.056** | **.378** | **.770^**^** | **1.000** |  |  |
|  | Sig. (2-tailed) | **.330** | **.294** | **.829** | **.838** | **.839** | **.456** | **.145** | **.896** | **.002** | **.203** | **.064** | **.064** | **.144** | **.037** | **.026** | **.224** | **.001** | **.076** | **.043** | **.803** | **.083** | **.000** |  |  |  |
|  | N | **22** | **22** | **22** | **17** | **17** | **17** | **17** | **20** | **20** | **20** | **22** | **22** | **22** | **17** | **17** | **17** | **22** | **22** | **22** | **22** | **22** | **22** | **22** |  |  |
|  | | | | | | | | | | | | | | | | | | | | | | | | |  |  |
|  | | | | | | | | | | | | | | | | | | | | | | | | |  |  |
